# Committing to Place: The Potential of Open Collaborations for Trusted Environmental Governance

**DOI:** 10.1371/journal.pbio.1002081

**Published:** 2015-03-05

**Authors:** Claire Waterton, Stephen C. Maberly, Judith Tsouvalis, Nigel Watson, Ian J. Winfield, Lisa R. Norton

**Affiliations:** 1 Department of Sociology, Lancaster University, Lancaster, United Kingdom; 2 Centre for Ecology & Hydrology, Lancaster Environment Centre, Library Avenue, Bailrigg, Lancaster, United Kingdom; 3 School of Sociology and Social Policy, University of Nottingham, Nottingham, United Kingdom; 4 Lancaster Environment Centre, Lancaster University, Lancaster, United Kingdom; King’s College London, UNITED KINGDOM

## Abstract

Conventional modes of environmental governance, which typically exclude those stakeholders that are most directly linked to the specific place, frequently fail to have the desired impact. Using the example of lake water management in Loweswater, a small hamlet within the English Lake District, we consider the ways in which new “collectives” for local, bottom-up governance of water bodies can reframe problems in ways which both bind lay and professional people to place, and also recast the meaning of “solutions” in thought-provoking ways.

## Abstract 2

This article considers how a willingness to experiment with new forms of participation for environmental management can pay off in ways that can make a difference to people and place.

This Perspective is part of the Public Engagement in Science Series.

## Introduction

In this article, we reflect on our involvement in a cross-disciplinary participatory initiative around a problem of lake water management to explore important issues in public engagement. Lakes provide diverse and essential ecological goods and services, and are extremely sensitive to natural and human induced perturbation at local to global scales. Diffuse and point sources of nutrient enrichment of water bodies resulting from the impacts of agricultural land use and human habitation constitute a considerable challenge for lake and catchment management worldwide [1]. This article considers the ways in which new “collectives” for local, bottom-up governance and management of such water bodies and their catchments can reframe these problems in ways that bind both lay and professional people to place, and also recast the meaning of “problem-solving” in thought-provoking and challenging ways.

The place in which we consider these issues is Loweswater, a small hamlet and lake with the same name, within the English Lake District. The lake has, since the early 2000s, experienced regular blooms of potentially toxic cyanobacteria (blue-green algae), caused primarily by phosphorus enrichment [2]. Conventional means of tackling this problem (scientific research and regulatory interventions from 2001 onwards) had produced little impact by the time the three-year research project discussed in this paper began in 2007 (Box 1). The problem was characterised by considerable uncertainty, complexity, and conflict regarding causes, consequences, corrective actions and responsibility, and seemed intractable. This project was conceived to work on the specific problem of phosphorus enrichment in Loweswater while also practically experimenting with new ways of carrying out participation in environmental decision making. We highlight five aspects in this article regarding how the experiment:

- Brought together participants with different backgrounds, knowledge, and roles;
- Challenged traditional approaches to creating knowledge and opened up new avenues of inquiry and research;
- Encouraged controversy and questioning by stakeholders about how they could best manage the environment at Loweswater as a group;
- Shed new light on the obstacles and challenges faced by institutions involved in land and water management;
- Promoted actions for the more effective management of the lake and its catchment.

**Box 1. Pre-existing Governance Arrangements for Land and Water at Loweswater**

Despite being a small catchment, arrangements for the management and governance of the land and water in Loweswater were complex and involved a wide range of actors, including individual land managers and both non-government and government organisations. The statutory responsibilities of the latter for the catchment focused around; i) land (Natural England; NE), ii) water (Environment Agency; EA), and iii) the landscape, including the economic and social wellbeing of local communities (Lake District National Park Authority; LDNPA). These responsibilities included both regulatory powers to penalise practices deemed environmentally damaging (EA) and funding to enhance ecological conditions of both land and water (NE and EA). Despite its statutory responsibilities, the LDNPA had limited planning powers to effect environmental change in the catchment. Catchment land owners, tenants, and managers included the National Trust (NT) and a small number of farmers and local residents.

Prior to the mid-2000s, although there was some collaboration between farmers, residents, the EA, and the NT around the nutrient enrichment of Loweswater and the presence of cyanobacteria in the lake, the institutions involved were acting in relatively insulated ways that excluded the Loweswater community. Scientists studied the lake, and the EA, which was responsible for implementing the (then) new and legally binding European regulatory framework (EU Water Framework Directive 2000) gathered data. However, a wider sharing of knowledge was lacking; a narrow framing of the problem predominated, and interactions between agencies and local people were limited and wary. Mistrust, blame, and lack of communication were common between the actors concerned. Meanwhile, the cyanobacterial blooms continued to occur regularly.

## The Loweswater Care Project

### Participants

From 2004 onwards, local residents, farmers, small business owners, the NT, environmental agencies, ecologists from the Centre for Ecology and Hydrology (CEH), and social and natural scientists from Lancaster University began to work together. This formed the basis of the project that started in 2007 during which the participants created a new “social mechanism,” named the Loweswater Care Project (LCP; Box 2). The LCP intended to experiment with alternative approaches to those conventionally adopted in the United Kingdom to deal with issues of diffuse and point pollution and, specifically, to improve existing arrangements for the management and governance of land and water in Loweswater, which seemed very fragmented and therefore ineffective at the time.

**Box 2. The LCP—How It Worked**

The LCP took on the form of a heterogeneous group of people. Members were not preselected, making this a very open forum. The LCP met 15 times for a full evening (5:30 p.m. to 9:00 p.m.) roughly every two months over a period of three years. It typically attracted between 25–35 participants, including 3–6 natural or social scientists from Lancaster University or CEH Lancaster, 2–5 agency representatives from NE, the NT, the LDNPA, the EA, and local residents, business owners, and farmers, among others. The agenda for each meeting was driven by LCP participants, and there was not a single strong “leader” of the group. Rather, the group worked collectively, generating ideas and future proposals from within. Meetings were usually chaired by Lancaster University or CEH researchers or a Loweswater farmer employed one day per week on the research project as a “community researcher.”

### New Avenues of Thinking

The idea behind the LCP built on the insights of a workshop that suggested that it would be useful to make much more effort to share and integrate a wide range of perspectives on “the problem” [3]. This was consistent with ongoing trends towards interdisciplinary collaboration and participation across domains of expertise in other areas of environmental management. Other examples come from catchment management [4]; flooding [5]; and seafood monitoring and fisheries management [6]. But the LCP wanted to do more than simply enhance existing politics by “bringing in” different people, things, and perspectives into a preexisting situation. Rather, the aim was partly to change the nature of the way in which stakeholders engaged with the problem at hand [7]. This implied struggles about what was at stake (and for whom and what) in the controversial situation of cyanobacteria continuing to bloom at Loweswater.

### Contested Knowledge

There was a sophisticated level of acknowledgement among LCP participants that there was not one single “problem” signalled by the presence of the algae. Consequently, the project sought to open up debates about how multiple issues could be framed collectively to take better account of alternative perspectives, experiences and forms of knowledge (Box 2).

### Challenges for Institutions

Previously, regulatory institutions and the NT were seen by some local farmers and businesses as “the opposition” as they largely used a “regulatory stick” to try to improve ecological quality in the lake (Box 1). Through LCP discussions, participants gained a much better appreciation of the constraints and difficulties faced by different actors in the catchment. However, it was not straightforward for the institutions to find staff time to engage with the LCP and, more particularly, to identify obvious institutional approaches to address the issues raised by the LCP.

### Underlying Principles

Social science researchers contributed a framework to maintain an open, critical, and questioning style of engagement within the LCP. Gaining new knowledge about the catchment was encouraged by all participants; but so was an intense questioning around any “facts” that were brought into the forum for discussion. This approach was designed to avoid some of the drawbacks of participatory frameworks that tend to focus too quickly on solutions, which gloss over the complexities of problems [8]. To this end, it was suggested in initial LCP meetings that the LCP adhere to a simple set of principles [9] such that:

- There is not a single “right” way of understanding nature;
- All knowledge and expertise needs to be debated;
- Uncertainties in knowledge need highlighting and accepting;
- New connections are valuable;
- Doubt and questioning needs to be extended to all the LCP’s representations, including scientific representations.

Applying these principles in practice was an experiment in reversing, in a particular time and place, the commonplace acceptance that problems such as phosphorus pollution can be deciphered by scientific expertise alone. It was an experiment in creating a new basis for action and management. In the terms of one contemporary analyst of science and technology, it was an experiment that prevented “science” from taking the place of “politics” [9].

LCP participants were keen to create a situation where both lay and expert practitioners could engage in the making of a more modest and provisional kind of knowledge that would support a more socially engaged way of managing water quality issues at Loweswater [5]. What this implied was a highly critical orientation to fact-making about the cyanobacteria, including facts derived from “objective scientific methods,” such as modelling. So, for example, when natural scientists Lisa Norton and Stephen Maberly presented to the LCP the results of their modelling work that attempted to connect terrestrial and aquatic mechanisms controlling phosphorus loads and lake response, much of the discussion and questioning focused around modelling itself. For example, what it can and can’t achieve, which parameters and inputs are included or left out, and what modelling results mean for on-the-ground realities. As such, science was conducted in ways that allowed for and encouraged critiques and evaluations of the ways scientific results and knowledge are acquired.

This kind of commitment to questioning often generated new lines of inquiry and collective work. For example, during 2007–2010, small projects, many undertaken by local people, were initiated and funded by the LCP. The results of these projects came back to the forum for further questioning and critique. As participants began to collect samples, to monitor different pathways of nutrient input, they began to question prior sampling techniques. As such, the LCP was bringing about a “coproduction” of knowledge and action [10].

### Impacts and Outcomes on Place and Beyond

During the project, participants of the LCP, building on the better relationships between different people and organisations, sought to identify practical actions that could be taken locally to reduce nutrient loads and consequent cyanobacterial blooms. For example, the NT, in consultation with farmers, funded work to clear vegetation from the stream that drained the lake to alleviate flooding and nutrient input from adjacent fields; NE provided advice about potential funding for joint catchment agri-environment scheme approaches; farmers and local residents successfully improved their waste management following a study by a local resident on the state of all the septic tanks in the catchment, which showed they made a significant contribution to the phosphorus load to the lake; and a survey of phosphorus levels in the soil by an agronomist employed by the project allowed farmers to reduce fertiliser applications in some fields, saving money and reducing nutrient loads to the lake. At a larger scale, the LCP disseminated its approach to other community-led groups, to local Rivers Trusts and, via local interactions and a workshop for national representatives, to organisations with responsibility for managing our environment. Some of these are currently working to align participatory collectives, like the LCP, with their existing responsibilities. The LCP provided inspiration for officials at the UK’s Department for Environment, Food, and Rural Affairs (Defra) who were planning the subsequent launch of the Catchment-based Approach (CaBA), which now has coverage across England [11].

There were also valuable place-based impacts and benefits for Loweswater itself. For example, building on the success of the LCP, Defra funded a follow-up project designed by Loweswater residents with resources from their Catchment Restoration Fund (CRF), which has allowed the local community to institute further practical initiatives. These include methods to reduce algal populations in the lake directly using ultrasonics, several farm-based initiatives which include using shared machinery to improve the percolation of water and nutrients into compacted soil, relocating cattle outside of the catchment (for a farm with inadequate facilities), improving slurry storage facilities to reduce slurry volume, and fencing wetland areas and streams to prevent cattle incursion. Some of the monitoring initiated by the project has been continued collaboratively between local residents and the EA. Although lakes take a long time to recover from nutrient enrichment, there are encouraging signs that water transparency is improving and chlorophyll concentrations are declining. The LCP and its successor programme funded under the Catchment Restoration Programme are contributing back to this place.

## Concluding Remarks

Between 2007 and 2010, the LCP opened up the issue of cyanobacteria in Loweswater to include discussion about seemingly distant, or even global, issues such as the possible impact of global climate change on lakes, the effects of the regulatory politics of the European Union, and the future of upland farming and rural societies in the modern world. Acknowledging the true complexity of the problem made it possible for all involved to see more clearly that the chronic ongoing pollution problem at Loweswater resulted from multiple, interacting stressors and multiple consequences within ecosystems and communities. Although at times this led to a loss of certainty and control around the boundaries of this problem and which actions might therefore be practicable, a gain was a sense that the search for absolute knowledge of the problem may not be the answer, in any case [12]. The LCP demonstrated that subtly changing the philosophical basis of knowledge-making within participatory initiatives can allow for more integrated and open-ended ways of governing complex socioecologies like Loweswater. This requires people from diverse backgrounds to create the knowledge and commit to the particularities of place, while recognising that place-based knowledge and action needs constantly to be challenged, kept open and carefully linked to frameworks of governance and the political arena. This is not just a search for solutions that have local buy-in, even though it may actually provide many of these (see “outcomes” above): it is, perhaps more importantly, a particular, located, way of working together on the kind of ongoing, chronic pollution problems characteristic of our times.

## Abbreviations

NE: Natural England
EA: Environment Agency
LDNPA: Lake District National Park Authority
NT: National Trust
LCP: Loweswater Care Project
Defra: Department for Environment, Food, and Rural Affairs
CaBA: Catchment-based Approach
CRF: Catchment Restoration Fund
CEH: Centre for Ecology and Hydrology
